# The prevalence and impact of *Babesia canis* and *Theileria* sp. in free-ranging grey wolf (*Canis lupus*) populations in Croatia

**DOI:** 10.1186/s13071-017-2106-8

**Published:** 2017-04-04

**Authors:** Ana Beck, Doroteja Huber, Adam Polkinghorne, Andrea Gudan Kurilj, Valerija Benko, Vladimir Mrljak, Slaven Reljić, Josip Kusak, Irena Reil, Relja Beck

**Affiliations:** 1 Department of Veterinary Pathology, Faculty of Veterinary Medicine, University of Zagreb, Vjekoslava Heinzela 55, 10000 Zagreb, Croatia; 2 Centre for Animal Health Innovation, University of the Sunshine Coast, 90 Sippy Downs Drive, Sippy Downs, 4556 Brisbane, Australia; 3 Internal Diseases Clinic, Faculty of Veterinary Medicine, University of Zagreb, Vjekoslava Heinzela 55, 10000 Zagreb, Croatia; 4 Department of Biology, Faculty of Veterinary Medicine, University of Zagreb, Vjekoslava Heinzela 55, 10000 Zagreb, Croatia; 5 Department for Bacteriology and Parasitology, Croatian Veterinary Institute, Zagreb, Savska cesta 143, 10000 Zagreb, Croatia

**Keywords:** Grey wolf, *Canis lupus*, Croatia, *Babesia canis*, *Theileria capreoli*, Necropsy, Cytology, Histopathology, Genotyping

## Abstract

**Background:**

*Babesia* spp. and *Theileria* spp. are important emerging causes of disease in dogs. Alongside these domesticated hosts, there is increasing recognition that these piroplasms can also be found in a range of wild animals with isolated reports describing the presence of these pathogen in foxes (*Vulpes vulpes*) and captive grey wolves (*Canis lupus*). The prevalence and impact of these infections in free-ranging populations of canids are unknown. To gain a better insight into the epidemiology and pathogenesis of piroplasm infections in free-ranging grey wolves, pathological and molecular investigations into captive and free-ranging grey wolves in Croatia were performed.

**Results:**

The carcasses of 107 free-ranging wolves and one captive wolf were the subjects of post-mortem investigations and sampling for molecular studies. A blood sample from one live captured wolf for telemetric tracking was also used for molecular analysis. PCR amplification targeting the 18S RNA gene revealed that 21 of 108 free-ranging wolves and one captive animal were positive for *Theileria/Babesia* DNA. Subsequent sequencing of a fragment of the 18S RNA gene revealed that 7/22 animals were positive for *Babesia canis* while the other amplified sequence were found to be identical with corresponding 18S rDNA sequences of *Theileria capreoli* isolated from wild deer (15/22). Haematological and cytological analysis revealed the presence of signet-ring shaped or pear-shaped piroplasms in several animals with the overall parasite burden in all positive animals assessed to be very low. Pathological investigation of the captive animal revealed fatal septicemia as a likely outcome of hemolytic anaemia. There was little or no evidence of hemolytic disease consistent with babesiosis in other animals.

**Conclusion:**

Importantly, the presence of *B. canis* in free-ranging grey wolves has not been described before but has been reported in a single fox and domestic dogs only. That *B. canis* infections cause disease in dogs but have little impact on wolf health possibly suggests that the wolf is the natural and the domestic dog is a secondary host. Surprisingly, the frequent finding of *Theileria capreoli* in wolves suggests that this *Theileria* species is not restricted to ungulates (cervids) but commonly infects also this carnivore species. Nevertheless, the potential role that these asymptomatically infected animals may play in the dispersal of these pathogens to susceptible sympatric species such as domesticated dogs requires further investigation.

**Electronic supplementary material:**

The online version of this article (doi:10.1186/s13071-017-2106-8) contains supplementary material, which is available to authorized users.

## Background

Piroplasms are tick-borne haemoprotozoal parasites able to infect almost all mammalian species. The two main genera of piroplasms infecting wild and domestic animals are *Babesia* and *Theileria*, with both traditional morphological methods and more recent molecular methods leading to the discovery and (re-)classification of new species among these hosts [1–3]. A diverse range of wildlife species have now been described as hosts for these piroplasms including mammalian species such as lions, lynx, panthers, elephants, giraffes, antelope, buffalo, several deer species, raccoons, hyena, mongoose, rhinoceroses, and bird species such as seagulls and the kiwi [4, 5].

In Europe, two ‘large’ canine *Babesia* species, *B. canis* and *B. vogeli*, are the most frequently detected piroplasms in domestic dogs [1]. Two other ‘small’ canine piroplasms species, referred to as “Babesia vulpes”, “Theileria annae” and *Babesia* cf*. microti*, and *B. gibsoni*, have been confirmed by molecular methods in dogs [6–8]. Non-canine species, *B. caballi* and *T. equi*, have been detected by PCR in symptomatic dogs in Spain and Croatia [9, 10]. These piroplasm species have been documented in Croatian symptomatic and asymptomatic dogs [10], while “B. vulpes”, “Theileria annae” and *Theileria* sp. have been confirmed in the Croatian population of free-ranging red foxes [11]. “Babesia vulpes” has also been molecularly confirmed in foxes from Spain, Hungary, Portugal, Italy and Bosnia and Herzegovina [12–17]. Unlike “B. vulpes”, *B. canis* has been reported only in a single animal during a study of the fox population in Portugal [14] and in a single fox in a similar survey in Bosnia and Herzegovina [17].

Beyond the limited data supporting the presence of piroplasms in foxes, information on the presence and prevalence of piroplasms in other wild carnivores such as grey wolves (*Canis lupus*) is extremely limited. Two reports have described the presence and pathogenic potential of *Babesia* in captive grey wolves from Poland and Hungary [18, 19]. In the former study, the diagnosis was based exclusively on clinical observations consistent with canine babesiosis (e.g. fever, splenomegaly, icterus, pigmenturia) that was resolved by imidocarb dipropionate treatment. Confirmatory diagnosis by detection of piroplasms in blood smear or molecular testing was not performed [18]. Erdelyi et al. [19] molecularly confirmed *B. canis* as a cause of death in two captive grey wolves suffering from the hemolytic disease. Another molecular epidemiological study conducted on an Italian free-ranging grey wolf population failed to detect piroplasms in any of the seven animals investigated [12]. Larger scale studies are otherwise absent since the distribution of the grey wolf populations in Europe is highly fragmented and separated by human habitat.

The Croatian population of free-ranging wolves is increasing in number and spreading from the south Mediterranean and central mountainous habitats to more urban north-west lowland geographical regions of Croatia [20]. In the current study, we performed a molecular survey of piroplasmids in free-ranging grey wolves with the sympatric presence of *Babesia canis*-infected domesticated canids and *Theileria* sp. in cervids, respectively. This paper also reports two clinical and post-mortem cases of asymptomatic *Babesia canis* infection in two young-adult, free-ranging grey wolves and a fatal infection with *B. canis* in a captive grey wolf.

## Methods

### Animals

Wolves have been protected by Croatian law since 9 May 1995 [21]. According to the Wolf Management Plan for Croatia [21], all carcasses of wolves found must be submitted to the Faculty of Veterinary Medicine, Zagreb to determine the cause of death following postmortem analyses. The range of the Croatian population covers about 18,000 km^2^ of the mountainous and Mediterranean region of Croatia, which corresponds to 31.8% of the total Croatian territory [20]. In addition, the Croatian grey wolf can also be sporadically found in an additional 6,000 km^2^ area (transitional habitat) which corresponds to 10.6% of Croatian territory [20]. During the period of this study, the grey wolf population in Croatia was estimated to consist of 209 individual animals distributed in 50 packs, with a density of 1.7 wolves per 100 km^2^ [22].

**Fig. 1:**
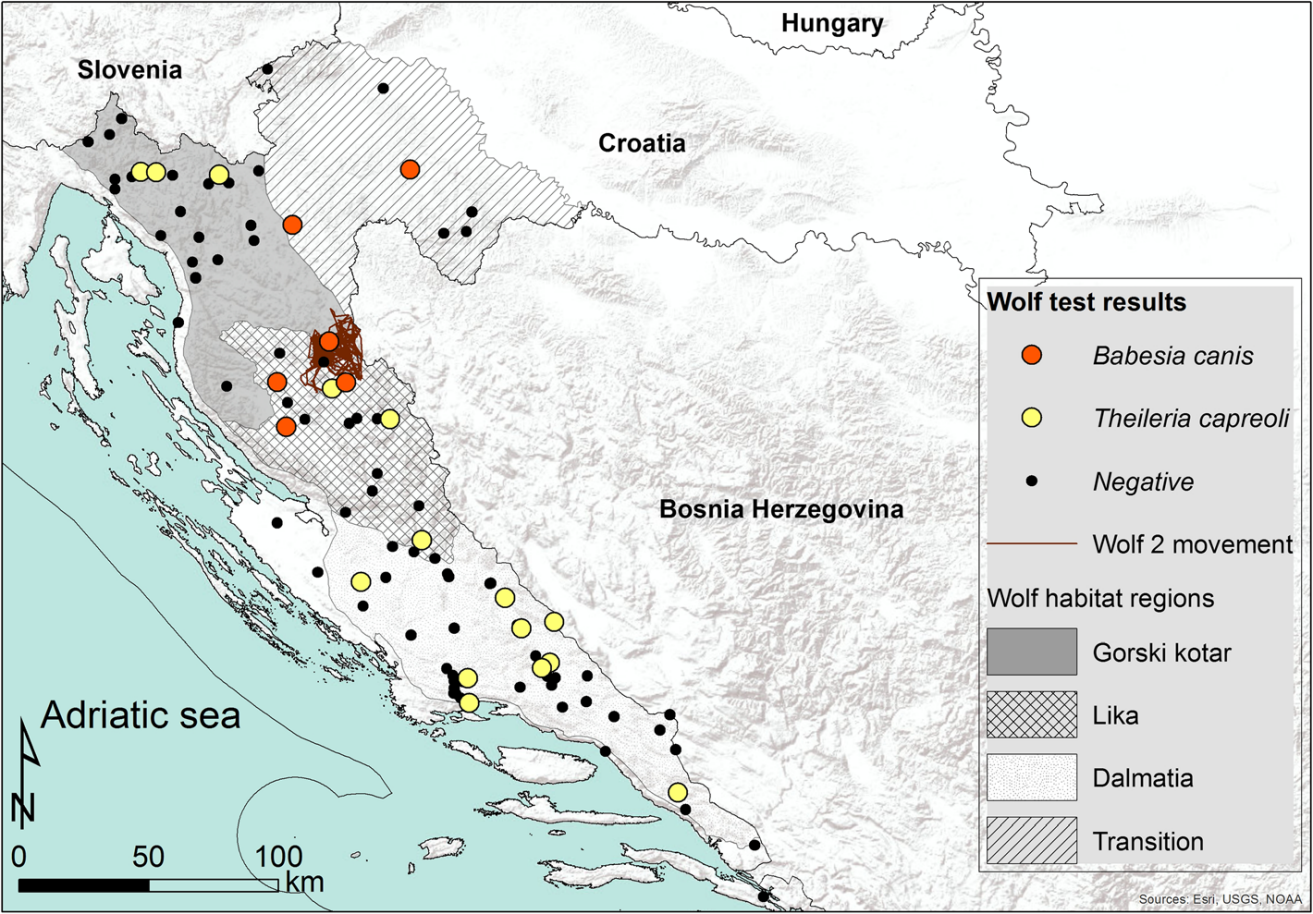
Map of Croatia with marked positions of all investigated wolves with molecularly confirmed parasites (Background map was created using ArcGIS® software by Esri. ArcGIS® and ArcMap™ are the intellectual property of Esri and are used herein under license. Copyright © Esri)

For each free-ranging wolf investigated, GPS coordinates of each carcass found were determined and recorded on a map of Croatia (Additional file 1: Table S1; Fig. 1). They originated from four geographical regions: Gorski Kotar, Lika, Dalmatia and the aforementioned ‘transitional habitat’. Gorski Kotar is a mountainous region with 63% of its surface covered by forest, mostly composed of deciduous and coniferous trees. The average altitude is 800 m with a moderate, rainy, continental climate zone [20]. Lika is a plateau in mountainous Croatia with similar forest cover and climatic conditions to Gorski Kotar [20]. Dalmatia is the southern part of Dinaric mountain range running from north-west to south-east. This region has a Mediterranean climate with vegetation mostly composed of bush, brush and evergreen plants. The transitional habitat is a region covered by deciduous forest and meadows with a moderate, continental climate [20].

A total of 108 free-ranging animals and one captive animal were investigated over a period of 20 years (1996–2015). Two out of 108 animals were found alive of which one was the animal (Wolf 109) held in captivity and was added to the study. A brief description of the prospectively sampled animals (Wolf 2, Wolf 107, Wolf 109) follows:

- Wolf 2: A male, 0.5 years old wolf was captured for telemetric research in Plitivička jezera national park, Lika region, Croatia, on 27 November 2014. The wolf was captured by live trap and immobilised with a combination of tiletamine hydrochloride and zolazepam hydrochloride (Zoletil 100©, Virbac, Virbac, Carros, France). During anesthesia, the animal was measured, equipped with a GPS tracking collar (Vectronic©, Berlin, Germany) and blood-sampled from the cephalic vein for routine hematologic and biochemical analyses. After completing all measurements, the wolf was left in a secure and silent location in the woods prior to recovery. For the following 12 months, the wolf was satellite-tracked with location recording in six-hour intervals, until the collar was automatically dropped from the animal’s neck.
- Wolf 107: A male 1.5-year-old wolf was brought to the Clinic of Veterinary Faculty, Zagreb after a car collision outside of the city of Glina in the transitional habitat on 18 November 2015 for emergency surgery. During surgery, blood was sampled for routine haematology and biochemistry. After clinical examination, the spinal cord injury was estimated to be too severe for survival. The wolf was humanely euthanised and delivered for necropsy.
- Wolf 109: A 17-year-old, male grey wolf born and kept in captivity in the Zagreb Zoo was delivered for necropsy on 26 May 2016. Seven months before death, the wolf was separated from the pack because of aggression towards other pack members, poor physical condition and poor sight. Three days before death, febrile disease with lethargy and anorexia was noted. Due to old age and the poor condition, the animal was humanely euthanised.

### Postmortem analysis

Carcasses of 107 free-ranging wolves (including Wolf 107) and one captive wolf (Wolf 109) were delivered for necropsy regardless of the degree of autolysis. Body measurements and weight were determined and the age was estimated by teeth inspection using the teeth wear technique [23]. Tissue samples from 26 non-autolytic carcasses were submitted for further routine histopathology. Brain, kidney, liver, lung, myocardium and spleen slices were fixed in 10% neutral formalin, dehydrated, paraffin embedded, cut to a thickness of 5 μm and stained with hematoxylin and eosin. Pieces of the brain, kidney, liver, lung, myocardium and spleen from each animal were frozen at -20 °C. Blood cloth, kidney, liver, myocardium, lung and spleen cut surface imprints were prepared, air-dried and stained using the May-Grünwald-Giemsa protocol. Due to post-mortal hemolysis, the number of intracellular and extracellular piroplasms was evaluated in tissue imprints in approximately 4,000 erythrocytes and their surrounding areas per each slide.

### Haematology and biochemistry

Blood smears were prepared from EDTA-sampled blood from Wolf 2 and Wolf 107, air-dried and stained with May-Grünwald-Giemsa. Parasite burden was determined by counting of parasites visible in high power fields (HPF) containing a total number of 4,000 erythrocytes (10 HPF). Hematologic analyses were carried out using an automatic haematological counter (Horiba ABX©, Micros, France). Serum biochemistry was made by an automatic biochemical analyser (OLYMPUS AU 640©, Olympus Chemistry Analyzer, Hamburg, Germany) for the basic metabolic panel using referral intervals for wolves according to published values [24]. A blood aliquot was frozen at -20 °C for molecular detection of pathogens.

### Molecular analysis

Frozen blood from 2 wolves (Wolf 2 and Wolf 107) and spleen samples from all 106 free-ranging wolves and the captive wolf from Zagreb Zoo (Wolf 109) were processed by the same protocol. DNA was extracted from 20 μg of tissue samples or 200 μl of blood using the DNA Blood and tissue kit (Qiagen, Hilden, Germany) in the automatic extraction system Qiacube (Qiagen). In each round of extraction, one sample of DNase/RNase-Free distilled water was included as a blind control for DNA extraction. To detect members of the genera *Babesia* and *Theileria*, a fragment (~560 bp) of the 18S rRNA gene was amplified and sequenced using the forward primer 5′-GTC TTG TAA TTG GAA TGA TGG-3′ and the reverse primer 5′-CCA AAG ACT TTG ATT TCT CTC-3′ [10]. After initial sequencing, a larger fragment of *Theileria* sp.-positive samples was re-amplified for the species conformation using a new set primers; forward 5′-AGT TTC TGA CCT ATC AG-3′ and the reverse primer 5′-TTG CCT TAA ACT TCC TTG-3′, that amplifies a 1,090 bp fragment of the 18S rRNA gene, as previously described [25]. *Babesia*-positive samples were further analysed with primers CRIPTOF 5′-AAC CTG GTT GAT CCT GCC AGT AGT CAT-3′ and CRIPTOR 5′-GAA TGA TCC TTC CGC AGG TTC ACC TAC-3′ that amplify complete 18S rRNA gene under conditions described by Caccio et al. [26]. Same primer sets were used for sequencing. PCR reaction mixtures of 20 μl were prepared to contain 10 μl G2 GO*Taq* master mix (Promega, Madison, WI, USA), 7.2 μl of DNase/RNase-Free distilled water (Qiagen), 0.4 μl of 10 pmol/μl each primer and 2 μl of sample. The successful amplification of PCR product was confirmed by capillary electrophoresis QIAEXEL (Qiagen) using a QIAxcel DNA Fast Analysis kit, alignment markers (DNA QXAlignmentMarker15 bp/3 kb) and QX DNA Size Marker 50–3,000 bp. Amplified PCR products were purified using EXOSAP-it® (USB® Products AffyInc., Ohio, USA) according to manufacturer’s instructions and sequenced in both directions (Macrogen, Amsterdam, the Netherlands). The resulting sequences were assembled using the SeqMan Pro software, edited with Edit Seq tools in Lasergene (DNASTAR, Madison WI, USA) and compared with available sequences using BLAST in GenBank.

## Results

### Pathological investigations of grey wolf carcasses

In total, 107 free-ranging grey wolf carcasses were dissected with the cause of death determined primarily by gross findings for 100 of these animals (Additional file 1: Table S1). The most common cause of mortality was trauma, followed by organ rupture and exsanguination in 98 free-ranging animals. Lesions were caused by car or train collision or by gunshot. Rarely trauma was induced due to intraspecific strife. Two animals that died due to rabies had unremarkable gross findings with a mild to moderate degree of teeth destruction and foreign bodies in the stomach. In seven wolves, the cause of death remained undetermined due to severe decomposition postmortem. None of the dissected wolves showed signs of hemolytic disease.

**Fig. 2:**
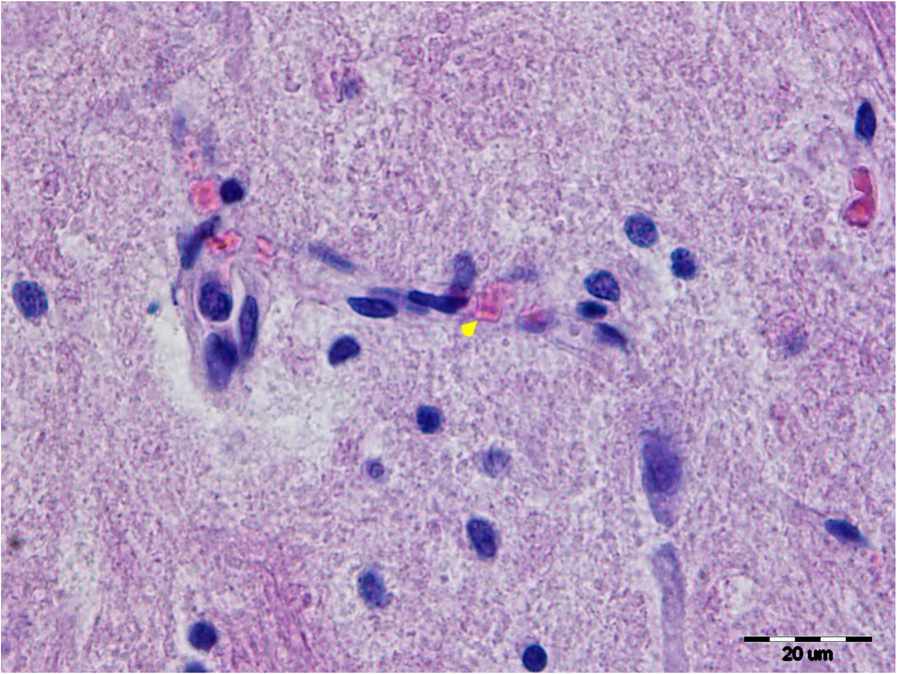
Round merozoites of *B. canis* (*arrowhead*) within one erythrocyte in cerebellar capillary. Hematoxylin and eosin staining, 1000× magnification. *Scale-bar*: 20 μm

**Fig. 3:**
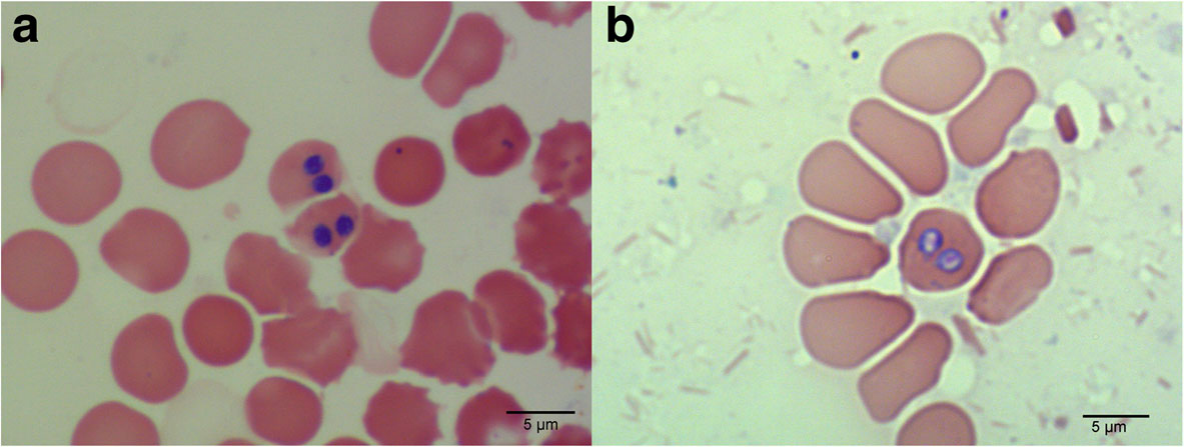
**a** Round *B. canis* piroplasms within erythrocytes of a blood clot obtained at postmortem from Wolf 107. **b** Erythrocytes parasitized by ring-shaped piroplasms on lung tissue imprint from Wolf 107. May-Grünwald-Giemsa staining, 1000× magnification. *Scale-bars*: 5 μm

Diffuse histiocytosis and mild erythrophagocytosis within the red pulp of the spleen were the single microscopic suggestions of hemolytic disease found in the wild Wolf 107, although round-shaped merozoites measuring 1.5 μm were seen within a few erythrocytes in the capillaries of the brain (Fig. 2) and the myocardium. In the postmortem cytological imprints from blood clots, kidney, liver and lungs, round-shaped merozoites measuring approximately 1.7 μm and rarely signet-ring shaped merozoites measuring 1.75 μm (Fig. 3) were present. The parasite burden in all postmortem samples was very low (approximately 20 piroplasms detected in 4,000 erythrocytes and their surrounding).

**Fig. 4:**
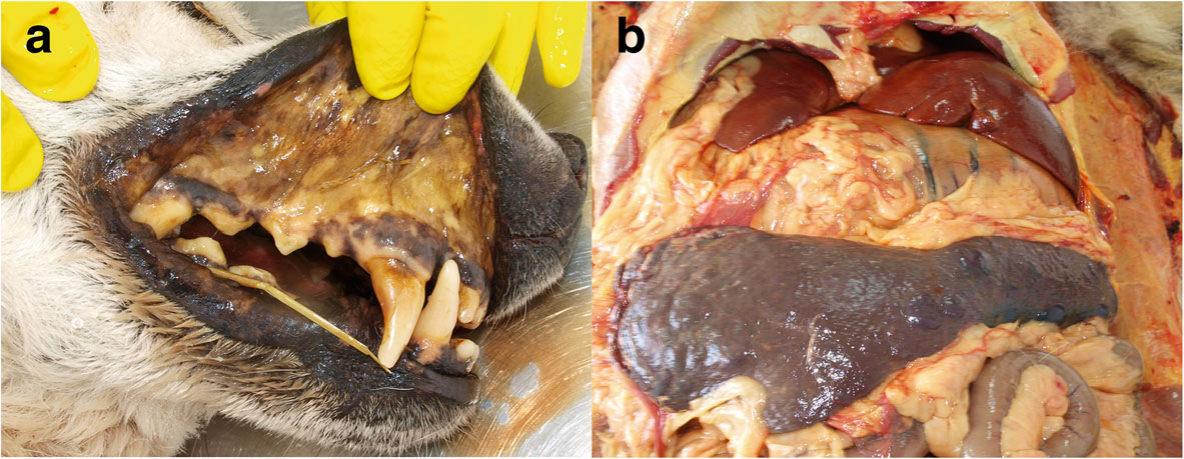
**a** Yellow discoloration of oral mucosa in Wolf 109. **b** Hepatosplenomegaly and yellow discoloration of abdominal fat tissue in Wolf 109

In cytological imprints from the kidneys, skeletal muscle and spleen from the captive wolf (Wolf 109), numerous round piroplasms measuring 1.7 μm were evident within erythrocytes or in the background of the cytological sample (approximately 350 piroplasms). Necropsy of the captive wolf revealed pronounced jaundice of mucosa (Fig. 4a), eye sclera and subcutaneous tissue (Fig. 4b). In this case, the cause of death was multiple organ dysfunction syndromes leading to suffocation due to diffuse hemorrhagic lung oedema development. Hepatosplenomegaly (Fig. 4b) developed due to severe capillary network dilation and blood plasma stasis. Systemic hypoxia was accented by extensive intravascular and extravascular hemolysis. Thrombotic lesions connected with sepsis were found in the spleen in the form of occlusive fibrinous thrombosis of splenic artery segments leading to focally extensive ischemic infarctions. Consequences of myocardial, pulmonary and hepatic disseminated intravascular coagulation were seen by disseminated areas of coagulative necrosis and haemorrhage. Both kidneys had undergone massive acute cortical necrosis most likely due to severe prerenal hypoxia. Discoloration of tubular epithelial remnants and formation of intratubular luminal casts developed because of hemoglobinuria and bilirubinuria, also visible by the formation of bile plugs in the liver.

### Haematology and biochemistry

Blood smears examined revealed signet-ring shaped or pear-shaped piroplasms within a few erythrocytes in Wolf 2 and Wolf 107. Signet-ring shaped piroplasms measured 2.1 μm in diameter. Pear-shaped piroplasms measured 1.8 μm in width and 4.4 μm in length.

Wolf 2 showed a mild normochromic normocytic anemia (4.3 × 10^12^/l; reference values 5.6–7.62 × 10^12^/l), slight thrombocytopenia (157 × 10^9^/l; reference values 194–567 × 10^9^/l) and an increase in the plasma value of creatinine kinase (2,802 U/l; reference values 95–1,315 U/l). Traumatised Wolf 107 showed normochromic normocytic anaemia (5.6 × 10^12^/l; reference values 6.36–8.19 × 10^12^/l). The biochemical profile revealed that only the concentration of the enzyme creatinine kinase (3,577 U/l; reference values 95–1,315 U/l) was elevated.

### Molecular detection of piroplasms

In the current study, samples from 108 free-ranging grey wolves and one captive grey wolf were screened for the presence of *Babesia/Theileria* DNA. Out of 108 free-ranging grey wolves, 21/108 (19.4%) were positive for *Babesia/Theileria* spp. DNA. The captive Wolf 109 was also PCR positive for *Babesia/Theileria* spp. DNA.

Direct sequencing of the PCR products of these positive cases was used to determine the infecting piroplasm species. *B. canis* was detected in three animals that have been studied in more detail in this investigation (Wolf 2, 107 and 109) as well as the carcasses of four free-ranging wolves (Wolf 32, 49, 51 and 104). Amplified DNA fragments of around 550 bp were found to represent an identical sequence which, following BLAST analysis, were identical to a *B. canis* isolate from a Croatian dog. Amplified fragments of 1,704 bp (GenBank accession number KY359360) from wolves 2, 107 and 109 were identical to isolate from a domestic dog from Croatia (AY072926). The remaining 15 PCR fragments of 550 bp from free-ranging wolves were also sequenced and, after BLAST search found to be 100% similar to the 18S rDNA sequence of two *T. capreoli* and the *Theileria* sp. 3185/02 isolate. For species confirmation, a larger fragment of 1,005 bp was successfully sequenced from six wolves, respectively, and deposited under accession number KY359359 in GenBank. All six sequences were identical to *T. capreoli* from wild Reeves’ muntjac (KJ451470), *T. capreoli* from roe deer (AY726011), and the *Theileria* sp. 3185/02 isolate from red deer (AY421708).

Mapping of the obtained data revealed a diverse distribution of *B. canis* and *T. capreoli* (Fig. 1). The putative *T. capreoli* positive samples were from wolves in all studied areas except the transitional habitat while *B. canis* was present only in the Lika region at around 600 m above sea level and in two wolves from the transitional habitat. The highest prevalence of both piroplasm species was detected in Lika (28%) with the transitional habitat having a similar prevalence (28.5%), albeit the only detected species was *B. canis* (Table 1). During 12 months monitoring of movement activity, *B. canis* infected Wolf 2 covered area of 563 km^2^ (Fig. 1). The putative *T. capreoli* piroplasms were the only ones detected in Gorski Kotar and Dalmatia with a similar prevalence of 13.6 and 16.7%, respectively (Table 1).

**Table 1:** Prevalence of infected free-ranging wolves from different geographical regions

Region	No. of animals	*Babesia canis* % (95% CI)	*Theileria* sp. % (95%CI)	Total % (95% CI)
Gorski Kotar	22	0	13.6 (75.0–33.3)	13.6 (4.8–33.3)
Lika	25	16.0 (6.4–34.6)	12.0 (4.2–29.9)	28.0 (14.2–47.5)
Dalmatia	54	0	16.7 (9.0–28.7)	16.7 (9.0–28.7)
Transitional habitat	7	28.5 (8.2–64.1)	0	28.5 (8.2–64.1)
Total	108	5.5 (2.5–11.6)	13.9 (8.6–13.9)	19.4 (13.0–27.9)

## Discussion

One of the most important canine vector-borne diseases in Croatia is babesiosis caused mostly by *B. canis* [10]. Apart from domesticated dogs, *B. canis* has been molecularly confirmed in the neighbouring countries of Bosnia and Herzegovina from a single fox [17] and two captive grey wolves from Hungary [19] as well as in a single red fox (1/91) from Portugal [14]. Since data on *Babesia* infection in free-ranging carnivores are scarce, we performed a molecular and pathological investigation of *Babesia* and *Theileria* species infecting the free-ranging population of wolves from Croatia to understand the prevalence, distribution and potential health impacts of these parasites on a wild canid population.

Molecular screening of free-ranging and captive grey wolves demonstrated that species of *Theileria* and *Babesia* could infect this host. To our knowledge, this study describes the first detection of *B. canis* in free-ranging wolves (5.5%) and a captive animal from Croatia (Wolf 109). As expected, based on analysis of 18S rRNA gene, the sequences were all identical to *B. canis* from Croatian dogs and similar to other European *B. canis* isolates [27, 28]. Interestingly, the prevalence of infected wolves from the Lika region and transitional habitat was almost identical. We were previously aware that the transitional habitat was endemic for *B. canis* infection in dogs [7], but the high prevalence in the more southern region of Lika was unexpected. The tick *Dermacentor reticulatus*, the only known vector of *B. canis*, was detected in wolves in the same region in 2009 (unpublished finding), fulfilling conditions for a continuous cycle of *B. canis* in wildlife and efficient spreading via infected ticks or infected animals [29]. A range of factors such as the ability of free-ranging wolves to cross a distance of several hundred kilometres [30] together with transovarial transmission of *B. canis* within *D. reticulatus* could facilitate the spread of the pathogen over long distances. This would appear to be confirmed in this study where Wolf 2, infected with *B. canis*, originated from the Lika region but moved over a large area and during a 12-month period covered 563 km^2^. Young individuals leaving the pack and searching for new territories are another aspect of dispersing pathogens [21, 30]. Young, dispersing wolves in search for new habitat can frequently be observed in Croatia, including areas of sporadic occurrence as well as the urban north-west lowland geographic regions [20].

Except for Wolf 2 which was still alive at sampling, all free-ranging wolves infected with *B. canis* died due to trauma or rabies. Regarding the clinical impact, based on clinical assessments and post-mortem investigations in this study, it seems that free-ranging wolves can tolerate infection with *B. canis*, the most pathogenic piroplasm species for canines in Europe, without the development of clinical signs of severe haemolytic disease. Supporting this assumption is the absence of clinical, haematological and biochemical changes consistent with canine babesiosis in Wolves 2 and 107. Even though piroplasms were proven in blood smears of these infected wolves, only discrete normochromic normocytic anaemia, thrombocytopenia and slight elevation of creatinine kinase were confirmed. Traumatised Wolf 107 probably showed normochromic normocytic anaemia and creatinine kinase elevation due to internal and external blood loss and muscle and spinal cord tissue rupture. Although in Wolf 2, slight anaemia and thrombocytopenia could relate to *B. canis* infection, elevated levels of serum creatinine kinase were expected due to skeletal muscle fibre damage caused by capture in a live trap with subsequent leakage of creatinine kinase [31].

Although only a sample size of one, the monitoring carried out in the 12 months following the capture of Wolf 2 also suggests little or no impact on the movement of these otherwise healthy animals. In contrast to these findings in free-ranging animals, the captive wolf (109) infected with *B. canis* showed typical gross and microscopic lesions characteristic for canine babesiosis following post-mortem analysis. Infection with *B. canis* in dogs often results in a wide range of clinical presentations characterised by fever, lethargy, thrombocytopenia, anaemia, icterus, hemoglobinuria and multiple organ dysfunction syndromes with fatal outcome [32], consistent with postmortal findings of the captive wolf in this study. Although dogs can be asymptomatically infected, the number of such cases is very low [10]. It seems that free-ranging wild animals can tolerate *Babesia* infection better than their domesticated “relatives”. This phenomenon has been described in cheetahs, and domestic cats, where cheetahs infected with *Babesia lengau* stay asymptomatic while infection in domestic cats progress and cause lethal cerebral babesiosis and haemolytic anaemia [33, 34]. The observation suggests that healthy, chronically infected wolves may act as reservoirs for *D. reticulatus* tick-mediated transmission to *B. canis* “free” regions and animals including domesticated dogs. A similar reservoir capacity was confirmed in experimental infection studies of young coyotes (*Canis lantras*) with *B. gibsoni* where animals were found harbouring parasites for several months without clinical signs [35].

Another piroplasm, suggested to be *Theileria capreoli* by 18S rDNA sequence analysis, was an unexpected finding in the current study while also representing the first detection of this pathogen in grey wolves. The parasite was more frequently detected of the two piroplasms (13.9%), and it appears that it was homogeneously distributed in all three regions except the transitional habitat. The 18S rDNA sequences amplified in this study were identical to or similar to the sequence amplified from *Theileria* strains from red deer (3185/02; 100% similarity, AY421708) and roe deer from Spain (*T. capreoli*, 100% similarity AY726011), a red fox (3185/02, 99.9% similarity, HM212629) [11], red deer from Poland (ZS T04, 99.9% similarity, DQ520836) [36], and a *T. capreoli* strain from a wild Reeves’ muntjac from China (100% similarity) [37]. Based on this study, it appears that *T. capreoli* or closely related *Theileria* sp. ZS T04, *Theileria* sp. 3185/02 can infect not only wild cervids but also wild canids such as red foxes and wolves. *Theileria* sp. 3185/02 was confirmed in roe deer and red deer from areas where two wolves were harbouring the same parasite (unpublished data). Detection of *T. capreoli* DNA in *Ixodes ricinus* ticks from Italy [38] raises the question whether this tick species may act as vectors for this *Theileria* species. In the current study, two *Theileria*-positive wolves from Gorski Kotar were found that share the same ecological niche with roe deer where *I. ricinus* was the only tick species detected [39]. Other infected wolves originated from mountainous areas where *I. ricinus* is the dominant tick species [40]. Further studies will be required to establish the possible role of *I. ricinus* as a vector for this piroplasm amongst different wildlife species.

Previous studies have shown that deer or roe deer are commonly infected with *Theileria* spp. without showing clinical signs [30]. Similarly, based on the observations made in this study on necropsied animals, no pathogenic effects of the putative *T. capreoli* infection (e.g. hemolytic disease) consistent with theilerosis was observed in free-ranging grey wolves. Our observations strongly suggest that the putative *T. capreoli* infections in free-ranging wolves are subclinical.

## Conclusions

In the current study, we have found that the infection of free-ranging grey wolves with *B. canis* and *Theileria capreoli* is without clinical or pathological signs of babesiosis and/or theileriosis. Accordingly, our study revealed that a *B. canis*-infected wolf showed a large range of movement, which also highlights the potential ‘risk’ for these asymptomatically infected animals to be ‘spill-over’ vectors of this pathogen into sympatric populations of domesticated dogs within their home-range. In contrast, a single *Babesia canis* infected captive animal did show symptoms of babesiosis. In this context, it should be mentioned that it has been described that piroplasmid-infected asymptomatic wild animals may die when suffering stressful situations (e.g. captivation). Finally, *Theileria* sp. has been detected in wolves that displayed identity with the 18S rRNA gene fragment described of *T. capreoli* from different cervides. Our findings suggest a broader host specificity for *B. canis* as well as *T. capreoli* as previously assumed while the absence of pathology of piroplasmid infected free-ranging wolves suggests that this host species may be the natural host of the detected piroplasmid species.

## Additional file

Additional file 1: Table S1. Raw data for investigated wolves. (XLSX 27 kb)

## Abbreviations

CI: confidence interval
GPS: Global positioning system
HPF: high power fields
PCR: polymerase chain reaction
